# Rosmarinic Acid: A Potential Therapeutic Agent in Gastrointestinal Cancer Management—A Review

**DOI:** 10.3390/ijms252111704

**Published:** 2024-10-31

**Authors:** Karolina Czerwińska, Iwona Radziejewska

**Affiliations:** Department of Medical Chemistry, Medical University of Białystok, ul. Mickiewicza 2a, 15-222 Białystok, Poland; karolina.czerwinska@sd.umb.edu.pl

**Keywords:** gastrointestinal cancers, polyphenolic compounds, rosmarinic acid

## Abstract

Gastrointestinal cancers are still the leading cause of death worldwide. This is related, among other things, to the non-specific symptoms, especially in the initial stages, and also to the limited possibilities for treatment. Therefore, research is still being conducted to improve the detection of this type of cancer and increase the effectiveness of therapy. The potential application of natural compounds in cancer management deserves special attention. In the group of such products, there are polyphenolic compounds that reveal, e.g., anti-oxidative, anti-carcinogenic, anti-inflammatory, anti-diabetic, and neuroprotective properties. One of these polyphenols is rosmarinic acid, commonly found in plants such as the Boraginaceae and Nepetoideae subfamilies of the Lamiaceae (mint) family. A number of studies have considered the positive effects of rosmarinic acid in the treatment of many cancers, including gastrointestinal ones such as oral, stomach, pancreas, colon, and liver cancers. The main aim of this paper was to summarize the mechanisms of action of rosmarinic acid in gastrointestinal cancers.

## 1. Introduction

Cancers are one of the leading causes of disease incidence and mortality worldwide, especially when it comes to gastrointestinal ones, which affect the digestive system. Generally, these cancers are considered the major diseases affecting human health. The development of the cancer process is provoked by genetic and environmental factors. It is estimated that about 50% of cancer events are caused by dietary habits and social behaviors [1]. According to data prepared by the Global Cancer Observatory, the number of cancer deaths around the world in 2022 was approximately 9.7 million, and the incidence was about 20 million. According to forecast data, it is estimated that the incidence by 2050 may increase to 35 million. The group of gastrointestinal cancers includes oral, gastric, pancreas, colon, and liver cancers. These cancers occupy leading positions in terms of the incidence and mortality of malignant tumors [2,3,4]. The reason for this is that most patients are diagnosed at the late stage of the disease due to the lack of characteristic symptoms of the disease in its initial stages. Ongoing studies aim to enhance the detection and therapy outcomes of these types of cancer [5]. There are various treatment strategies for digestive tract tumors. These include surgery and postoperative adjuvant chemo- and radiotherapy. Chemotherapy is a standard approach but has serious limitations, such as poor water solubility, low tumor targeting ability, adverse effects, and chemoresistance. One more alternate choice of treatment seems to be immunotherapy. This has gained preference recently mainly because of its ability to alter the tumor microenvironment by supporting anti-tumor immune responses, improving the effectiveness of self-effector T cells and antigen-presenting cells. Such a treatment offers advantages over other therapy modalities, like reduced side effects [6].

Due to the reasons mentioned above and also because of an increase in the resistance of mammalian tumors towards existing anticancer drugs, there is a great need to develop alternative novel medications [7]. It is well known that diet can play a crucial role in cancers. Epidemiological studies have indicated that decreased global cancer risks might be related to the regular consumption of a high-fiber, low-fat diet including a significant intake of vegetables and fruit [8,9].

Recently, special emphasis has been placed on exploring the impact of natural compounds, particularly plant-derived polyphenols, at different stages of cancer development. Polyphenols are broadly present in beverages and foods of plant origins (e.g., tea, wine, coffee, vegetables, fruit species). They refer to a wide group of plant secondary metabolites, which can be small molecules or greatly polymerized large compounds. The quality and quantity of polyphenols in food depend on many factors, such as plant genetics, growing conditions, harvest maturity, or post-harvest handling [10]. Chemically, polyphenols are characterized by the presence of at least one phenol unit with one or more hydroxyl groups linked to it [11,12]. They represent a wide range of structural diversity and include phenolic alcohols, phenolic acids, and many more molecules with hydroxyl groups on aromatic rings [13]. Polyphenols play a crucial role in both plant biology and human health. Plants synthesize polyphenols to defend themselves against infection and protect themselves from stress [14]. They reveal broad and multifaceted bioactive properties. Polyphenolic compounds represent a promising and less invasive alternative to conventional treatment possibilities, with the potential to augment the efficacy of standard therapeutic approaches [15,16,17,18,19,20,21]. Recently, the role of dietary polyphenols in repairing epigenetic alterations in cancer cells, including DNA methylation and histone modifications, has been reported. Research has also demonstrated the ability of polyphenolic compounds to modulate gene expression at the epigenetic level and presented their molecular targets in cancers [14]. Thus, polyphenols are proposed to prevent or reverse cancer-related epigenetic disorders [22,23].

One example of such a compound is rosmarinic acid (RA), presenting low acute toxicity [24]. Its beneficial effects have been revealed in the treatment of various cancers, including gastrointestinal ones like oral, stomach, colorectal, pancreas, and liver cancers [25,26,27,28,29,30,31,32]. There have also been reports indicating the gastro-protective effects of RA in ethanol-induced gastric lesions in vitro and in animal models [24,33]. Recently, several studies have revealed that rosmarinic acid is able to reverse cancer resistance to first-line chemotherapeutics and also plays a protective role against the toxicity induced by chemotherapy and radiotherapy, mainly because of its scavenger capacity [34,35]. Thus, the main aim of this study was to summarize the mechanisms of RA’s action in the cancers mentioned above.

## 2. Rosmarinic Acid (RA)

### 2.1. Structure and Origin

**Figure 1:**
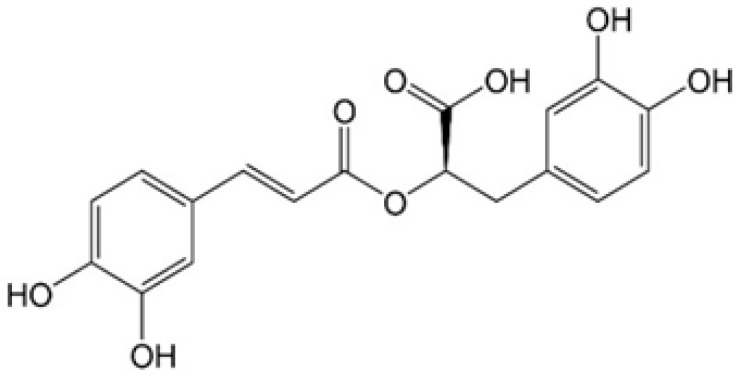
The structure of rosmarinic acid.

Chemically, rosmarinic acid, with the molecular formula C_18_H_16_O_8_, is a polyphenolic compound and a partly water-soluble ester of caffeic acid and 3,4-dihydroxyphenyl lactic acid with two catechol structures, which gives it a molecular mass of 360 kDa (Figure 1). RA is widely present in over 160 species, commonly found in plants from the Lamiaceae and Boraginaceae families, including the following genera: *Coleus* Lour., *Lavandula* L., *Melissa* L., *Mentha* L., *Origanum* L., *Salvia* L., *Thymus* L., *Zataria* Boiss., *Borago* L., and *Symphytum* L. These plants are very popular worldwide and are applied in everyday life in herbal preparations and teas, and as herbs, cooking spices, vegetables, and fruits [26,28,34,36,37]. Rosmarinic acid was first successfully isolated in a pure form from rosemary in 1958 [38].

Phytochemicals, including RA, are derived from plants’ seeds, fruits, roots, and leaves. It has been reported that the content of rosmarinic acid varies among species of the same genus, subspecies of the same species, and even samples collected in different seasons of the year [31,39]. There are also other factors that have an important role in the number and content of active ingredients in medicinal plants, such as the organ used, harvest time, growth stage, and planting conditions [40].

### 2.2. Biosynthesis, Extraction, and Purification of Rosmarinic Acid

The phenylpropanoid pathway is the primary pathway for the biosynthesis of rosmarinic acid and other phenolic compounds [38]. Initially, L-phenylalanine is transformed into t-cinnamic acid via phenylalanine ammonia lyase. Cinnamate 4-hydroxylyase, a cytochrome P450 monooxygenase, then converts t-cinnamic acid into 4-coumaric acid through hydroxylation at the 4-position. Coenzyme A ligase acts on 4-coumaric acid to produce 4-coumaroyl-CoA, which serves as a donor for hydroxycinnamate in a compound derived from tyrosine. In the first step of the L-tyrosine reaction, pyridoxal phosphate-dependent tyrosine aminotransferase uses 2-oxyglutarate as a co-substrate, yielding the transaminated form of 4-hydroxyphenylpyruvic acid and glutamate. This stage marks the junction of rosmarinic acid, tocopherol, and plastoquinone biosynthesis. Hydroxyphenylpyruvate dioxygenase acts on 4-hydroxyphenylpyruvate, converting it into homogentisic acid, a precursor for tocopherols and plastoquinones. For rosmarinic acid biosynthesis, 4-hydroxyphenylpyruvate is converted by NAD(P)H-dependent hydroxyphenylpyruvate reductase into 4-hydroxyphenyllactic acid, specifically the R (+) stereoisomer of hydroxyphenyl lactate. While hydroxyphenylpuryvate reductase can reduce 3,4-dihydroxyphenylpyruvate, it has low affinity. Alternatively, 3,4-dihydroxyphenylalanine can supply a 4-hydroxyphenyl lactate moiety. These two compounds, 4-coumaroyl-CoA and 4-hydroxyphenyllactic acid, are the final substrates for rosmarinic acid synthesis catalyzed by rosmarinic acid synthase. Through hydroxylation catalyzed by two cytochrome P450 monooxygenases, 4-coumaroyl-4′-hydroxyphenyllactic acid ester is converted into rosmarinic acid [38].

RA can be isolated from plant material in many ways. These include heat-assisted, ultrasound, and microwave extraction [36,41]. Up to now, standard solvent extraction has been the major pathway for extracting various phytochemicals from many plants [42]. For instance, Escalada et al. (2011) effectively produced ethanoic and aqueous extracts of *M. officinalis* containing mainly rosmarinic acid. The authors revealed that ethanol exhibited better extraction effectiveness toward phenolic compounds [43]. Modified or new protocols have recently been reported to provide better recovery rates and purities. Saad at al. (2021) demonstrated a robust procedure for the selective extraction of rosmarinic acid by applying molecularly imprinted polymers. The extraction of RA from *Rosmarinus officinalis* L. extract with a higher recovery rate and purity was achieved [44]. In another study, Dil et al. (2020) reported a procedure for the recovery of rosmarinic acid from medicinal plant extracts by applying chitosan–zinc oxide nanoparticles as a biocompatible nanocomposite [45].

### 2.3. General Action of Rosmarinic Acid in Tumor Prevention and Treatment

Rosmarinic acid displays a wide range of valuable, multifaceted, health-enhancing properties. There are many studies indicating that RA exerts anti-inflammatory and antioxidant effects, as well as inhibiting cell proliferation, migration, and adhesion and inducing apoptosis, suggesting that it could be beneficial in preventing tumor growth and metastasis [31,32,34,46].

Oxidative stress is induced by the excessive gathering of free radicals, which can be involved in a variety of cancers’ development. It was reported that rosmarinic acid has the power to remove these free radicals by enhancing antioxidant enzymes such as superoxide dismutase, catalase, glutathione peroxidase, and non-enzymatic antioxidants [28,47]. Other authors demonstrated that the acid intensifies the Nrf2/HO-1 antioxidant system to downregulate NLRP3 and IL-1β in cancer cells [48]. In other studies, the anti-inflammatory effects of RA were revealed by the downregulation of COX-2, NF-kB, and ERK1/2 [28,49]. Moreover, Achour at al. (2021) proved the inhibition of colorectal cancer development by rosmarinic acid’s action due to its antioxidant and anti-inflammatory effects [9]. It was also demonstrated that RA prevents tumor development via the inhibition of DNA damage as a result of the acid’s potential antioxidant ability [50].

Cell cycle arrest and the inhibition of proliferation have been applied in the therapy of several cancers [28]. Rosmarinic acid is able to achieve such aims mostly by the upregulation of p53 and p21 and the downregulation of cyclins D1, E, and B1 [51,52,53,54]. In other reports, the authors revealed that RA is able to directly regulate cell proliferation-associated targets such as EGFR, MARK4, and MCM7 [55,56,57,58,59,60].

The induction of apoptosis is one more excellent target in cancer therapy. Rosmarinic acid’s action was demonstrated to induce the expression of apoptosis-related factors such as Bax, caspase-3, and caspase-8 and suppress the expression of anti-apoptotic proteins like Bcl-2 and PARP in different types of cancer cells [16,27,51,52,61,62]. It was also reported that the PI3K/Akt and NF-kB signaling pathways are influenced by rosmarinic acid to induce apoptosis [16,63]. In a study carried out by Messeha et al. (2020), the authors revealed that RA stimulated apoptosis via the upregulation of the TNF and TNF receptor superfamily [64]. Moreover, another study demonstrated that rosmarinic acid combined with anti-MUC1 monoclonal antibody induced pro-apoptotic proteins such as p53, Bax, Bad, and caspases-3, -8, and -9 [65].

It was revealed that epithelial–mesenchymal transition (EMT), which is a characteristic transformation that occurs in invasive tumor cells, can also be affected by rosmarinic acid’s action. This acid inhibits EMT through the upregulation of E-cadherin, downregulation of N-cadherin, and inhibition of MMPs’ activity. In this way, RA is able to impair the invasive ability of cancer cells [27,62,66,67].

Reducing the invasion and metastasis of tumor cells is considered another important aim of cancer therapy. There are studies demonstrating the power of rosmarinic acid to inhibit these processes. Metalloproteinases of the extracellular matrix facilitate invasion and metastasis. RA was observed to decrease cells’ invasive abilities by inhibiting MMP-2 and MMP-9 in several tumor cell lines [16,27,51,63]. Furthermore, tumors’ invasion ability is suppressed by the action of rosmarinic acid via decreases in Akt phosphorylation and MMP activity [56,62,66,67]. Apart from this, RA was demonstrated to inhibit cancer metastasis via the VEGF and IL-8 signaling pathways [68,69,70], as well as by the NF-kB ligand/TNF receptor superfamily member 11a/osteoprotegrin pathway [70].

Downregulation of the glycolytic pathway can be also a goal of anticancer therapy. The Warburg effect is attributed to the glycolytic production of ATP and is considered a universal hallmark of most cancer cells [71]. It was reported that rosmarinic acid suppresses the Warburg event through the IL-6/STAT3 inflammatory pathway and the inhibition of HIF-1α, a transcription factor affecting the glycolytic path [72,73].

In addition, some reports demonstrated the potential of rosmarinic acid to act as a chemosensitizer via the modulation of specific signaling pathways. It is acknowledged that drug resistance is the main reason for chemotherapeutic treatment failure in many human tumors and is related to the type of cancer, stage, drug delivery, and individual condition of the patient [35,74]. For years, there have been indications to combine natural products with traditional therapies [75]. This is due to the fact that these natural compounds can be active towards a number of different molecular targets in cancer cells, impeding their growth and survival [76]. There are reports in which the authors stated that RA directly or indirectly intensifies the potency of established anticancer drugs, resulting in an improvement in their therapeutic effects [35,55,77,78]. Because of the presence of two catechol moieties, rosmarinic acid creates a polar molecule and is thereby able to penetrate lipid bilayers and save them from oxidation without changing their structure. Thus, RA reveals pro-oxidant and antioxidant power. The antioxidant capacity of rosmarinic acid is strengthened by its ability to remove hydrogen peroxide and free radicals [31,79]. Such an action enables RA to exhibit interactions with specific proteins known to be dysregulated in different cancer cells. In this way, rosmarinic acid reveals the potency to augment the power of many chemotherapeutics against chemoresistance in these cells [35]. A summary of the general action of rosmarinic acid is presented in Table 1.

It is worth emphasizing that rosmarinic acid has a very low toxicity and is quickly removed from the blood [80,81]. Among several clinical surveys conducted with RA-enriched dietary supplements, there were no reports of adverse reactions [28,82]. Only a few complaints, such as dry mouth, abdominal discomfort, itchy scalp, and headaches, have been reported [83].

Nevertheless, despite such promising results concerning rosmarinic acid’s action in different tumors, the mechanisms underlying the therapeutic activities of RA need further, wider investigations.

### 2.4. Preclinical and Clinical Studies on RA

In accordance with the outcomes of some preclinical pharmacological studies, the use of rosmarinic acid seems to be a promising strategy for cancer prevention and management [26,44]. It was reported that dietary supplementation of *O. stamineus* RA-enriched Nuvastatic TM enhanced oxidative damage and restored mitochondrial and cellular functions to reduce fatigue in patients with solid stage I–IV tumors. Furthermore, the neuroprotective effects of rosmarinic acid likely mitigated pain, lethargy, sleep loss, and other manifestations correlated with cancer-related fatigue in a Phase III trial (NCT04546607) [25]. Moreover, RA-containing extracts have been successfully applied in subjects with atopic dermatitis [84], knee osteoarthritis [83], and age-associated memory impairments [85].

## 3. Rosmarinic Acid in Different Types of Gastrointestinal Cancer—Mechanisms of Action

### 3.1. Oral Cancer (OC)

Oral cancer is ranked among the most prevalent human cancers globally. The overall five-year survival rate of this cancer is much lower compared to those for, e.g., breast and prostate cancers [51]. Since 2000, the incidence rate of this cancer has grown by 2% each year [86]. In the United States, oral cancer is becoming one of the most common cancers [79].

Recently, rosmarinic acid has been studied as a potential anticancer agent against human oral cancer cells. Luo et al. (2020) reported promising results demonstrating that RA was able to inhibit the proliferation of SCC-15 oral cancer cells. This antiproliferative effect was exerted via the induction of apoptosis and arrest of the cell cycle at the G2/M phase. The ratio of apoptosis-associated proteins Bax/Bcl-2 and the level of caspase-3 protein were shown to increase under rosmarinic acid treatment. Mitotic cell cycle arrest occurred due to the decrease in the cyclin B1 concentration caused by RA. Apart from this, rosmarinic acid reduced the MMP-2 and MMP-9 protein concentrations [51].

### 3.2. Colorectal Cancer (CRC)

Colorectal cancers rank as the third most frequent cancer by incidence and the second most dominant cause of cancer-associated deaths. It was estimated that in 2022, over 1.9 million new cases and 904,000 deaths occurred [2].

Rosmarinic acid seems to reveal potential anticancer effects toward colorectal cancer, acting on many steps of cancer development. Han et al. (2018) demonstrated that RA promoted cell death in metastatic CRC cells and suppressed their metastatic properties. It activated cycle arrest in the G0/G1 phase and apoptosis in colorectal cancer cells. Moreover, rosmarinic acid was able to inhibit the metastatic potential of CRC cells, including epithelial–mesenchymal transition, migration, and invasion, by the activation of AMPK. RA modulated EMT by the upregulation of an epithelial marker, E-cadherin, and downregulation of the mesenchymal markers N-cadherin, snail, twist, vimentin, and slug. Apart from this, rosmarinic acid suppressed the expressions of MMPs-2 and -9, ICAM-1, and integrin β1 [27,38].

In HT-29 human colon cancer cells, an RA treatment decreased N-cadherin expression and increased E-cadherin expression by TGFβ induction. It was also reported that rosmarinic acid inhibited the expression of MMPs-1, -3, and -9 and suppressed EMT through p38 MAPK/AP-1 signaling. The inhibitory effect of RA on EMT was diminished by miR-1225-5p knockdown [66].

In another study carried out by Jin et al. (2021), RA’s potency as an anti-tumor factor in colitis-associated colon cancer (CAC) was evidenced. Rosmarinic acid was able to reduce the colitis severity, inflammation-associated protein expression, tumor occurrence, and colorectal adenoma expansion. Moreover, it was observed that RA attenuated tumor growth by decreasing the expression of anti-apoptotic factors by the inhibition of TLR4-mediated NF-kB and signal transducer and activator of transcription 3 (STAT3) [87,88].

The anticancer action of RA in HT-29 colon cancer cells was also demonstrated through its inhibition of the expression of the proinflammatory gene COX-2, which is considered a risk factor in cancer development. Moreover, the authors revealed that rosmarinic acid reduced AP-1 and the cellular levels of ERK1/2 activation [49,89].

In another study, the authors observed that RA, with its suppressor effect on COX-2, in combination with ginsenoside Rg1, had an inhibitory outcome on the binding of PD-1 and PD-L1, which resulted in the further suppression of lung metastases of colon cancer [90].

Targeting Warburg metabolism has been reported to be a promising method for the treatment of colon cancer [72]. Xu et al. (2016), studying colorectal carcinoma, found that rosmarinic acid suppressed glucose consumption and lactate generation. The authors also demonstrated that RA reduced the expression of hypoxia-inducible factor-1α (HIF-1α), which influences the glycolytic pathway. Finally, it was concluded that rosmarinic acid could significantly regulate miR-155 and successively alter IL-6/STAT3 signaling, resulting in the suppression of inflammation in the tumor microenvironment and a possible anti-Warburg effect [73].

Moreover, in another study, the authors demonstrated that RA inhibited the cell adhesion, migration, and invasion of Ls174-T human colon carcinoma cells mainly via extracellular signal-regulated protein kinases (ERKs). Rosmarinic acid suppressed the activities of EGFR and VEGFR and also inhibited the nuclear translocation of NF-kB by pAkt and pERK de-phosphorylation. In addition, RA decreased the level of reactive oxygen species (ROS) and repressed the activity of metalloproteinases-2 and -9. Interestingly, the authors claimed that rosmarinic acid, in a proper concentration, had great anti-tumor and anti-metastatic abilities similar to those represented by the chemotherapeutic vinblastine [26,27,36,91].

Furthermore, RA administration diminished tumor formation and aberrant crypt focus multiplicity and modified antioxidant status and oxidative stress markers in animal models of colorectal carcinoma induced by 1,2-dimethylhydrazine (DMH). Apart from this, rosmarinic acid attenuated the activities of fecal and colonic bacterial enzymes implicated in colorectal cancer progression. From these results, the authors proposed RA as a chemopreventive agent [92,93].

Another study demonstrated that rosmarinic acid was able to induce apoptosis in the HCT15 human colon carcinoma-derived cell line by inhibiting ERK phosphorylation [94]. Moreover, in another study, Xavier et al. (2009) observed apoptosis induced by RA in the human colon carcinoma-derived cell lines HCT15 and CO115, which have different mutations in the MAPK/ERK and PI3K/Akt signaling pathways. Rosmarinic acid reduced ERK phosphorylation in HTC15 but had no effect on Akt phosphorylation in CO115 cells [95].

It was also revealed that RA from rosemary extract induced apoptosis and inhibited cell proliferation in the HCT116 and SW480 colon cancer cell lines through the Nrf2/ARE signaling pathways [96].

In addition to the above, studies conducted with animal models demonstrated the chemoprotective effects of rosmarinic acid in rat colon carcinogenesis. RA reduced DNA damage and inhibited the formation of aberrant crypt foci [97]. In another study, Ilhan et al. (2022) reported similar protective effects, showing that rosmarinic acid limited azoxymethane-induced colon carcinogenesis [98]. Additionally, Karmokar et al. (2012) evidenced that chronic consumption of RA slowed intestinal adenoma in mice [99].

### 3.3. Gastric Cancer (GC)

Yearly, about 990,000 individuals are identified with gastric cancer globally, with approximately 738,000 deaths. Thus, GC ranks as the fourth most commonly occurring cancer and the second leading cause of cancer-related deaths [1].

There are many reports demonstrating that gastric cancer’s development can be influenced by the action of rosmarinic acid. It seems to be useful as a pharmacological support for prophylactic use to prevent gastric lesions [24]. Radziejewska et al. (2018) suggested the usefulness of RA as a complementary agent supporting gastric cancer treatment. The authors revealed the inhibitory effect of the acid on MMP-9 and TIMP-1 activity, which was correlated with increased collagen type I expression. Rosmarinic acid also decreased the expression of the tumor-associated carbohydrate antigens Tn, T, and sialyl Tn, as well as MUC1 mucin, the main carrier of the mentioned antigens [100]. In another report, the authors demonstrated the anticancer effects of RA alone and in combination with anti-MUC1 monoclonal antibody in AGS gastric cancer cells. The applied treatment inhibited the expression of cancer-related sugar structures such as Tn, T, sialyl Tn, sialyl T, and fucosylated sugar antigens, as well as the expression of enzymes participating in their formation: ppGalNAcT2, C1GalT1, ST6GalNAcT2, ST3GalT1, and FUT4. Rosmarinic acid also decreased the expression of Gal-3 participating in metastasis [65].

In another study, Han et al. (2015) proposed that RA might potentially be a therapeutic agent for suppressing the Warburg effect in gastric carcinoma. The authors demonstrated that rosmarinic acid is able to inhibit glucose uptake and lactate production, as well as inhibit HIF-1α suppression. RA is also able to decrease the expression of proinflammatory cytokines and miRNAs related to inflammation, suggesting that it may suppress the Warburg effect through an inflammatory pathway, such as IL-6/STAT3 [71].

Another report suggested the potential of rosmarinic acid as a multidrug-resistance-reversing factor in gastric cancer. The authors revealed that RA enhanced the chemosensitivity of SGC7901 gastric cancer cells resistant to 5-Fu by downregulating miR-6785-5p and miR-642a-3p and increasing FOXO4 expression. It was observed that regulation of the MDR-associated protein P-gp and pro-apoptotic Bax might also be involved in this chemosensitizing action of rosmarinic acid [77].

Chen et al. (2023) reported that rosmarinic acid applied to SGC-7901 gastric cancer cells induced apoptosis through the mitochondrial pathway. It inhibited cell viability, mobility, and Bcl-2 expression. Oppositely, the apoptosis rate, Bax, cytochrome C, and cleaved caspase-3 expression were induced by RA’s action. Further, the authors suggested that gastric cancer cells could be induced by rosmarinic acid to arrest their cell cycle in the G0/G1 phase [101].

Recently, there have been attempts to synthetize RA analogues (RAAs) and apply them as anticancer drugs. In a study carried out by Li et al. (2019), the authors demonstrated promising results concerning RAA-11, a synthetic analogue of rosmarinic acid, as the compound which was able to inhibit growth, proliferation, and colony formation in SGC-7901 human gastric cancer cells. Apart from this, it induced apoptosis by the EGFR/Akt/NF-kB pathway. From these results, the authors suggested RAA-11 as a potent agent for gastric cancer treatment [102].

### 3.4. Pancreatic Cancer (PC)

One more kind of cancer that can potentially be influenced by rosmarinic acid is pancreatic cancer. In 2022, there were about 511,000 newly identified cases of PC and 467,000 deaths. Pancreatic cancer ranks as the sixth leading cause of cancer-associated deaths, accounting for about 5% of global cancer fatalities [2]. The PC mortality rate is very close to its incidence rate, and about 90% of patients cannot be treated by surgery [103].

Zhou et al. (2022) proposed rosmarinic acid as an effective therapeutic agent in the treatment of pancreatic ductal adenocarcinoma (PDAC). The authors observed that RA induced G1/S cell cycle arrest and apoptosis by regulating the expression of P21, P27, CDK2, cyclin E, Bax, and Bcl-2. It was also revealed that the acid inhibited cell migration and invasion through E-cadherin and MMP-9. In addition, the authors demonstrated that rosmarinic acid suppressed tumor growth, presumably by inhibiting Gli1 in a proteasome-dependent manner [52].

In another study, the authors proposed rosmarinic acid as a promising candidate to inhibit human pancreatic cancer progression. They revealed a significant suppression of cell growth, invasion, and migration by RA’s action. Furthermore, the RA treatment triggered apoptosis and lowered the expression of EMT markers such as vimentin and N-cadherin [32].

In studies performed by Han et al. (2019), the authors demonstrated that rosmarinic acid exerted suppressing effects on cell viability, cell growth, invasion, migration, and epithelial–mesenchymal transition, as well as inducing apoptosis in the Panc-1 and SW1990 pancreatic cancer cell lines. It was suggested that these tumor-inhibitory outcomes revealed by RA were achieved via regulation of the miR-506/MMP2/16 signaling pathway [27,67].

### 3.5. Liver Cancer (LC)

Liver cancer is estimated to be the sixth most common cancer globally and is considered the third leading cause of cancer-related deaths worldwide. In 2022, there were almost 865,000 newly diagnosed cases of liver cancer and about 757,000 deaths [2].

There are reports that liver cancer can also be affected by rosmarinic acid. An et al. (2021) observed that RA decreased the proliferation rate, migration, and invasion of HepG2 hepatoma cancer cells. The authors also revealed an inhibitory effect of rosmarinic acid on MMP-2 and MMP-9, as well as an inducing effect on apoptosis. Bcl-2, an apoptosis suppressor protein, was downregulated, while the pro-apoptotic protein Bax and cleaved caspase-3 were upregulated. Apart from this, it was reported that HepG2 metastasis was inhibited by RA via the PI3K/Akt/NF-kB signaling pathway [63].

Chen et al. (2023) demonstrated that rosmarinic acid had a similar effect on SGC-7901 gastric cancer as on HepG2 liver cancer cells. Apoptosis was induced via the mitochondrial pathway. The expression of the anti-apoptotic factor Bcl-2 decreased, but that of Bax, cytochrome C, and cleaved caspase-3 increased. Liver cancer cells were induced by RA to arrest their cell cycle in the S phase. In addition, cell viability and mobility were also suppressed by rosmarinic acid’s action [101].

In another study, the authors revealed the similar anticancer potential of rosmarinic acid toward human HepG2 liver carcinoma cells. The induction of apoptosis exerted by RA’s activity was associated with a decrease in Bcl-2, an increase in Bax levels, and the activation of caspases-3 and -9. DNA fragmentation upon rosmarinic acid’s action was observed as well. Moreover, RA led to inhibited cell migration and invasion by liver cancer cells [104].

Wang et al. (2019) reported that rosmarinic acid suppressed proliferation, invasion, and tumor growth in the SMMC-7721 hepatocellular carcinoma cell line. The authors demonstrated G1 arrest and apoptosis induction. It was suggested that such effects were achieved by the inhibition of PI3K/AKT/mTOR signal pathway. They also revealed that RA could suppress tumor formation by SMMC-7721 cells in vivo [105].

## 4. Limitations of Rosmarinic Acid’s Applications

Pharmacokinetic studies applying orally administered rosmarinic acid reported its low oral bioavailability. This is mainly due to its limited solubility in water and low membrane permeability [36]. This ineffective permeability is especially due to the strongly acidic nature of RA [106]. Its bioavailability after oral administration is also low due to the instability of RA when it passes through the gastrointestinal tract [107]. To overcome these problems, new technical options for the administration of RA have been demonstrated. The incorporation of rosmarinic acid into cyclodextrins, nanoemulsions, phospholipid complexes, solid lipids, or chitosan nanoparticles to improve its bioavailability has been proposed [108,109,110,111,112].

## 5. Conclusions

**Figure 2:**
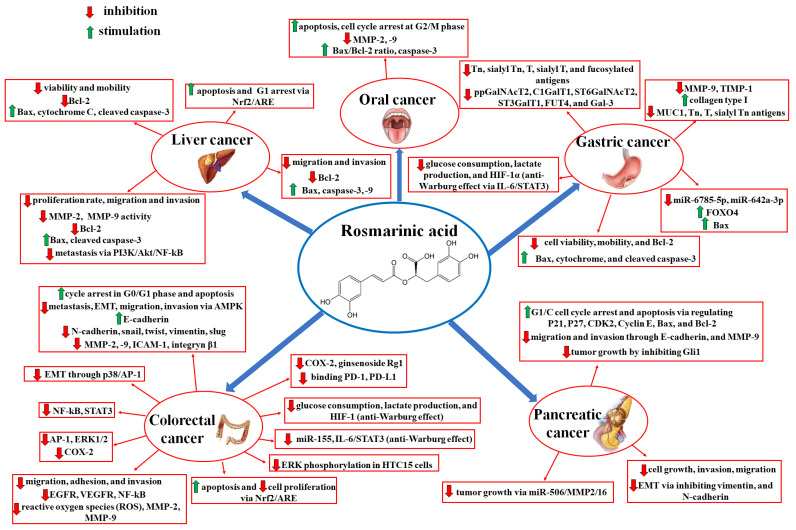
Summarized action of rosmarinic acid in oral [51], colorectal [26,27,36,38,49,66,72,73,87,88,89,90,91,94,95], pancreatic [32,52,69], gastric [65,71,77,100,101], and liver cancers [63,101,104,105].

Scientists focus on the significance of many herbal compounds in the treatment of many different types of cancers. As presented above, rosmarinic acid, commonly found in many medicinal plants, seems to be one such promising agent with the power to support the treatment of cancers, including gastrointestinal ones. Different signaling pathways can be affected by RA to reveal its anticancer potential (summarized in Figure 2). These research findings suggest that this acid is worth including in one’s everyday diet. However, it is worth emphasizing that the effectiveness of rosmarinic acid depends on its intake, stability, and bioavailability. As mentioned above, the latter factor for rosmarinic acid is quite low. Therefore, improvements to the dosage form and the development of chemical delivery systems are necessary for its anti-tumor applications. Further preclinical and clinical studies seem to be crucial to thoroughly clarify its therapeutic power.

## Figures and Tables

**Table 1:** General action of rosmarinic acid.

Biological Process	Effect of Rosmarinic Acid Action	References
Inflammatory and Oxidative Stress	RA shows anti-inflammatory effects by downregulating COX-2, NF-kB, and ERK1/2; reducing oxidative stress; and enhancing antioxidant enzymes (SOD, CAT, GPx). It also intensifies the Nrf2/HO-1 system to inhibit cancer cell markers.	[28,47,48,49]
Metastasis and Invasion	RA inhibits cell proliferation, migration, and adhesion; induces apoptosis; and prevents DNA damage, which contributes to the inhibition of tumor growth and metastasis. It reduces tumor cell invasion by inhibiting MMP-2, MMP-9, and Akt phosphorylation, and it decreases metastasis through the VEGF, IL-8, and TNF receptor pathways.	[16,27,31,32,34,51,68,69,70]
Cell Cycle Arrest	RA promotes cell cycle arrest and reduces cancer proliferation by regulating targets like p53, p21, and cyclins D1, E, and B1, as well as proliferation-related targets such as EGFR and MCM7.	[28,51,52,54,55,56,57,58,59,60]
Apoptosis	RA induces apoptosis by regulating factors such as Bax, caspases-3 and -8, and TNF receptors, while suppressing anti-apoptotic proteins like Bcl-2 and PARP in various cancer cells.	[16,27,61,62,63,64,65]
EMT	RA inhibits epithelial–mesenchymal transition by upregulating E-cadherin and downregulating N-cadherin and MMPs, reducing the invasive ability of cancer cells.	[27,62,66,67]
Glycolytic Pathway	RA inhibits the glycolytic pathway and Warburg effect in cancer cells via IL-6/STAT3 inflammatory pathways and the suppression of HIF-1α, reducing ATP production.	[71,72,73]
Chemosensitization	RA enhances the effectiveness of anticancer drugs by interacting with specific proteins and pathways, strengthening their therapeutic impact on chemoresistant cancer cells.	[35,55,76,77,78]

## Data Availability

No new data were created or analyzed in this study. Data sharing is not applicable to this article
